# Supplementary figures and images for: Burden of hemoglobinopathies and hemolytic anemias in the World Health Organization African region, 2000–2021: Findings from the Global Burden of Disease 2021 study
Source: PLOS Glob Public Health. 2025 Sep 22;5(9):e0005197. doi: 10.1371/journal.pgph.0005197 (PMC12453249; doi:10.1371/journal.pgph.0005197)

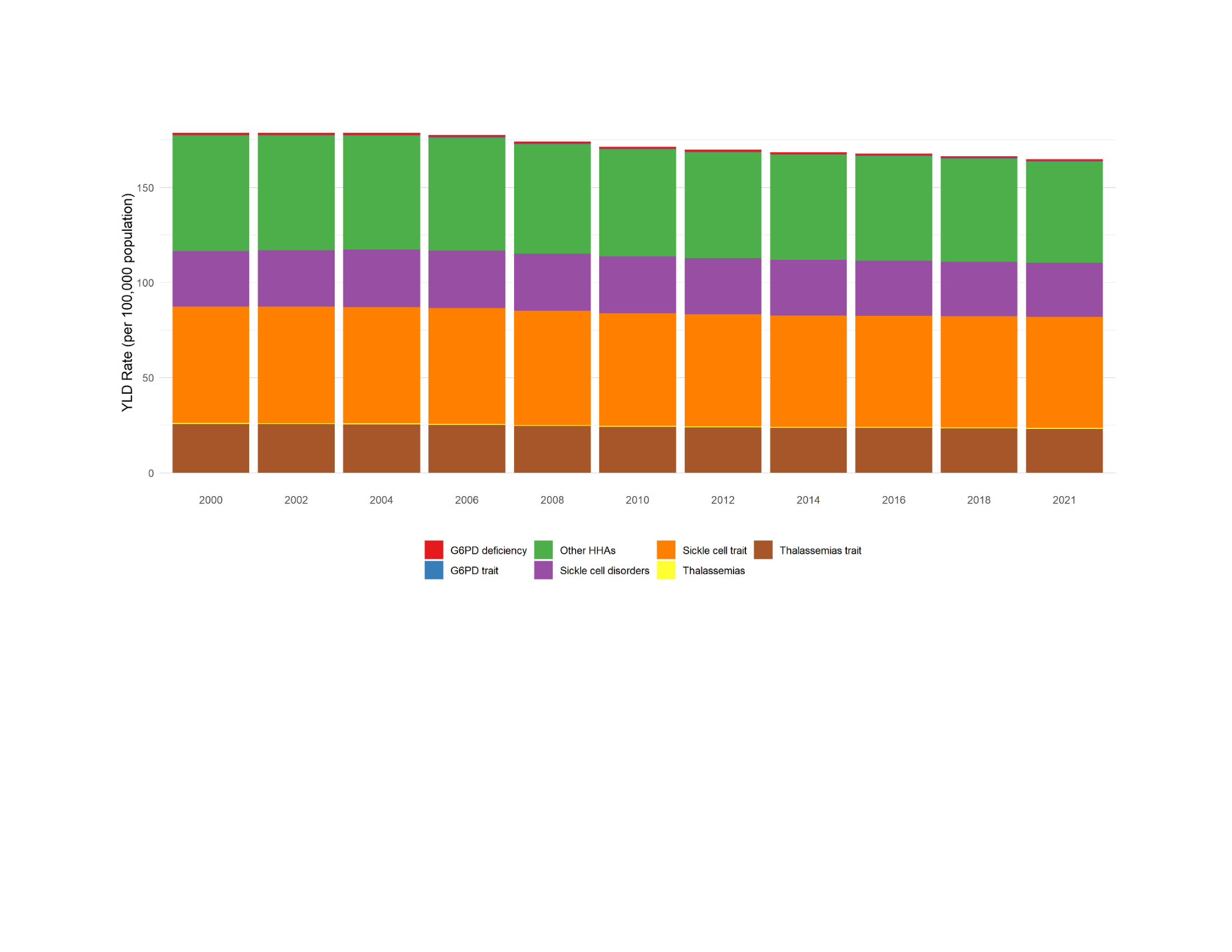

Supplement: S1 Fig — (TIFF) [file pgph.0005197.s001.tiff]

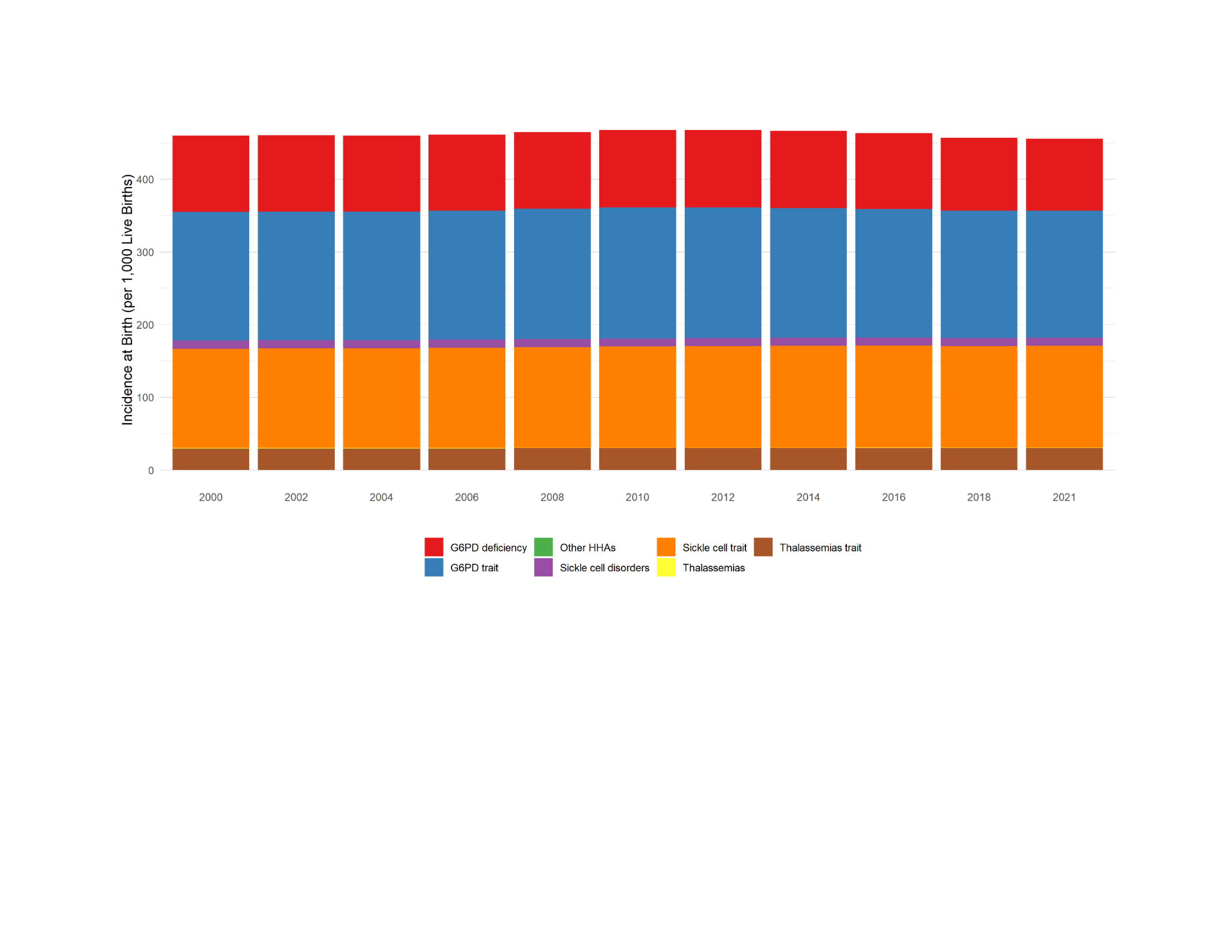

Supplement: S2 Fig — (TIFF) [file pgph.0005197.s002.tiff]

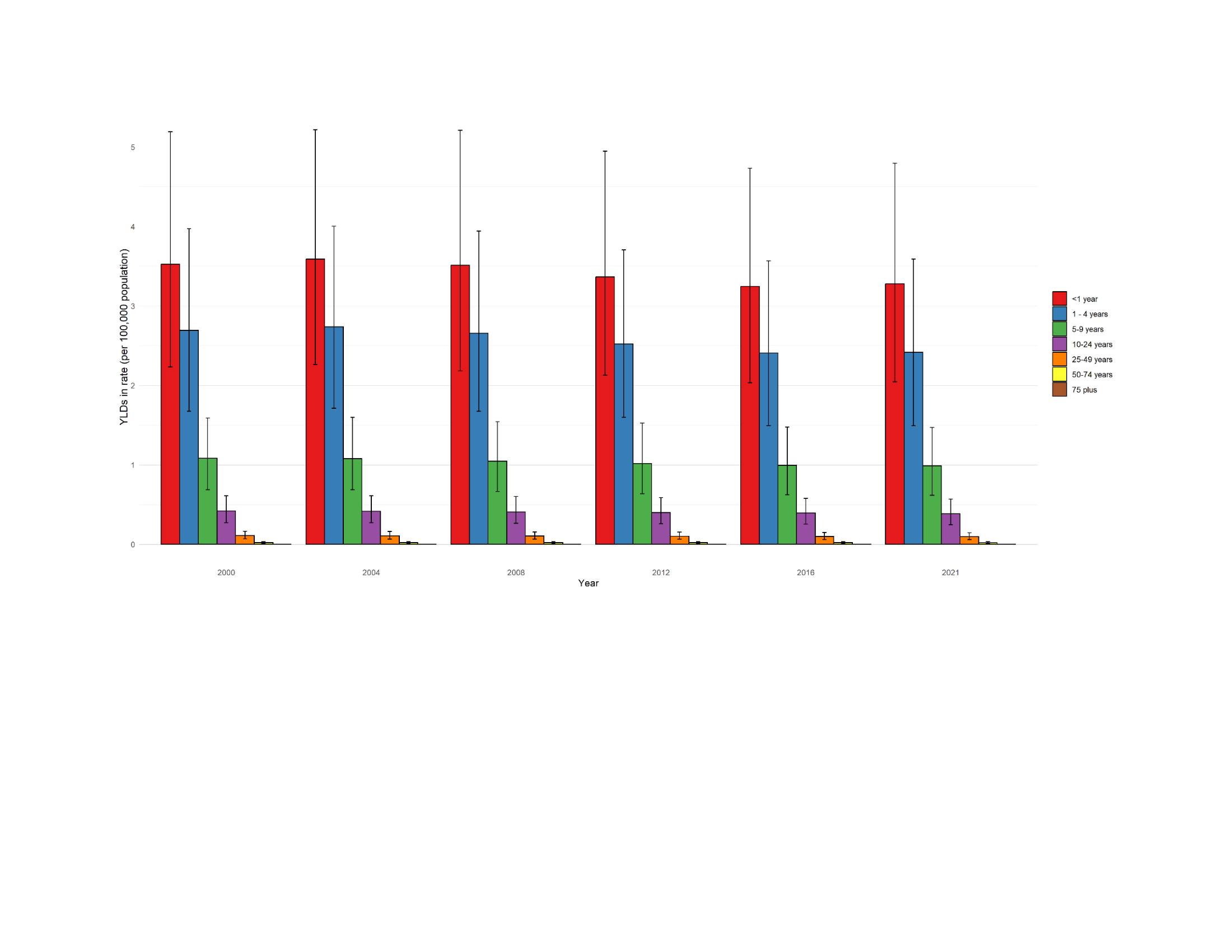

Supplement: S3 Fig — (TIFF) [file pgph.0005197.s003.tiff]

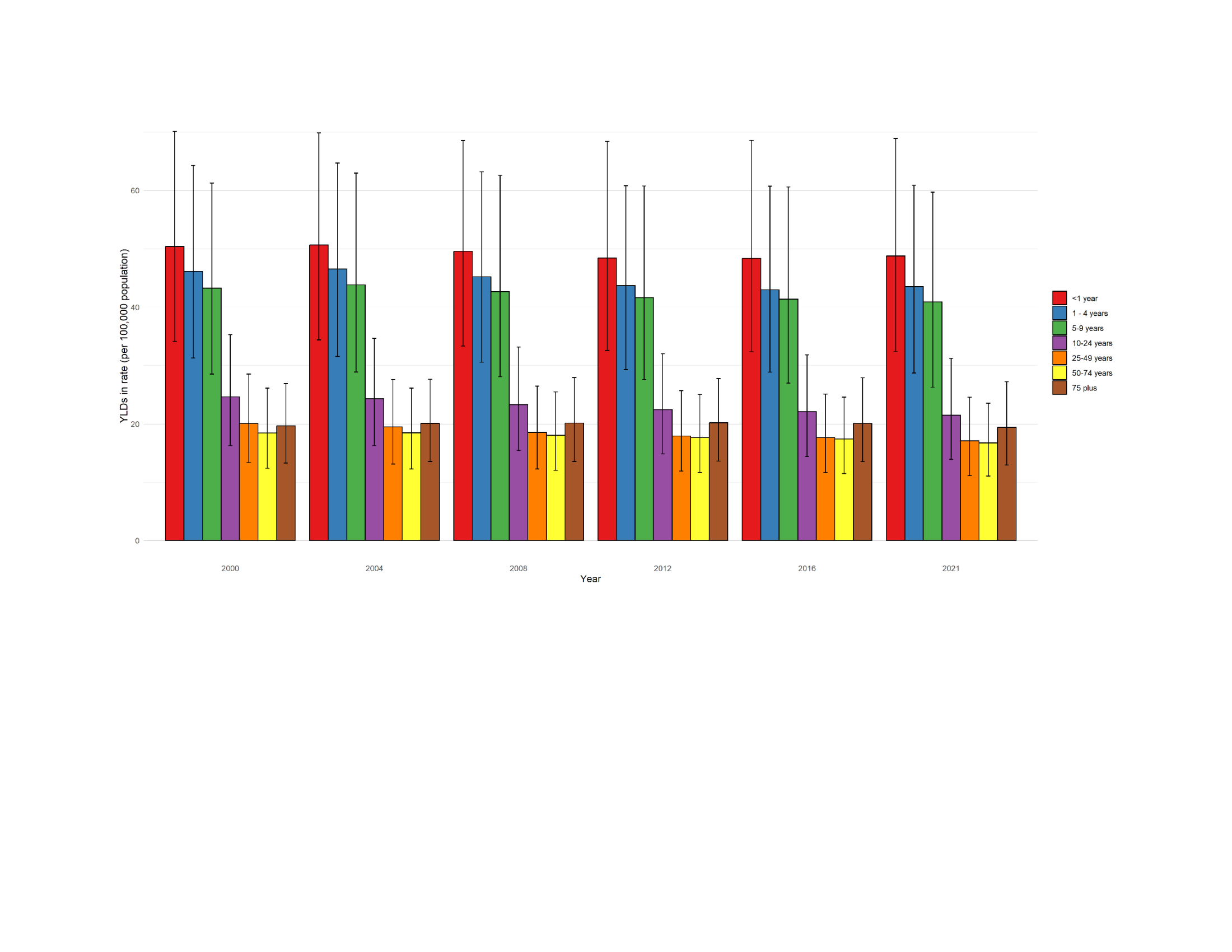

Supplement: S4 Fig — (TIFF) [file pgph.0005197.s004.tiff]

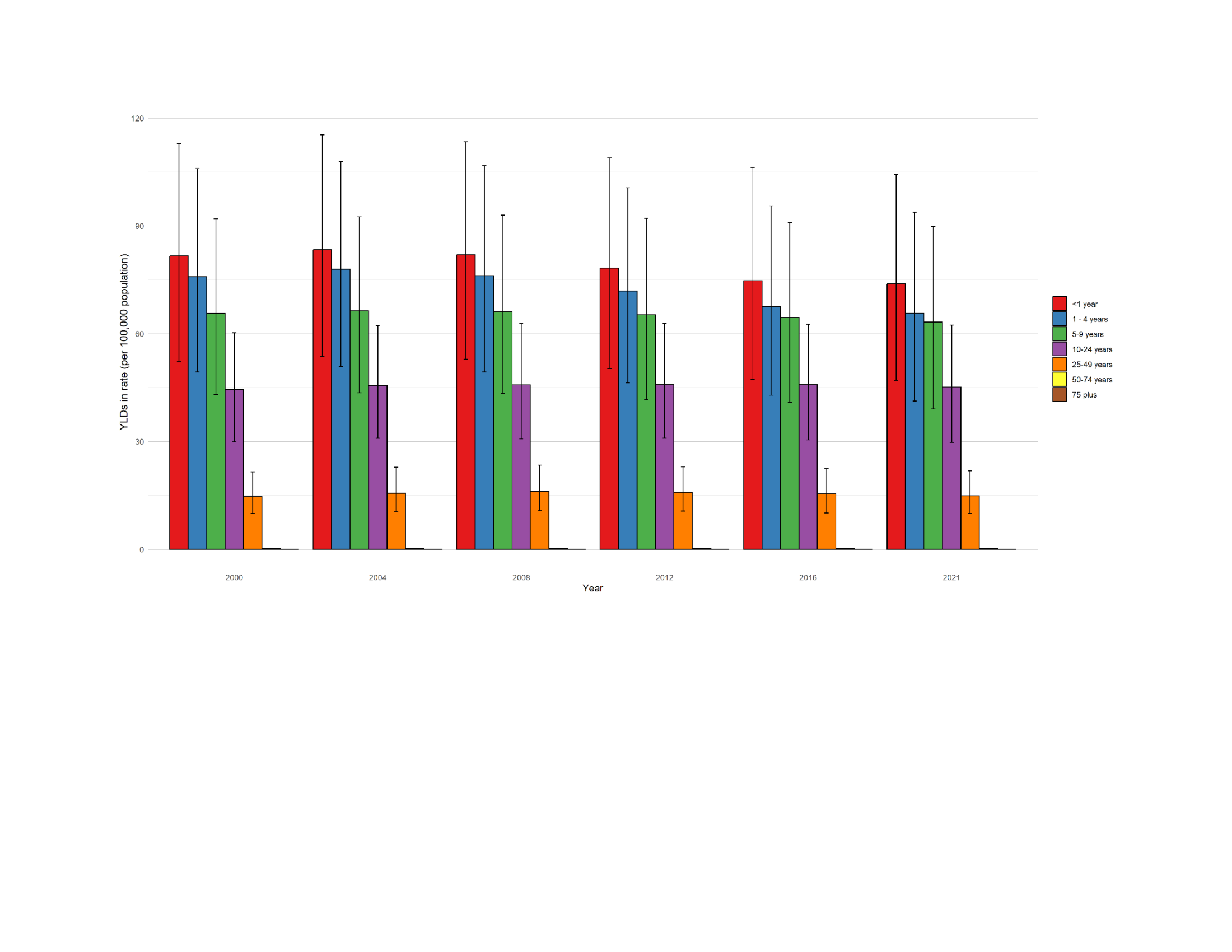

Supplement: S5 Fig — (TIFF) [file pgph.0005197.s005.tiff]

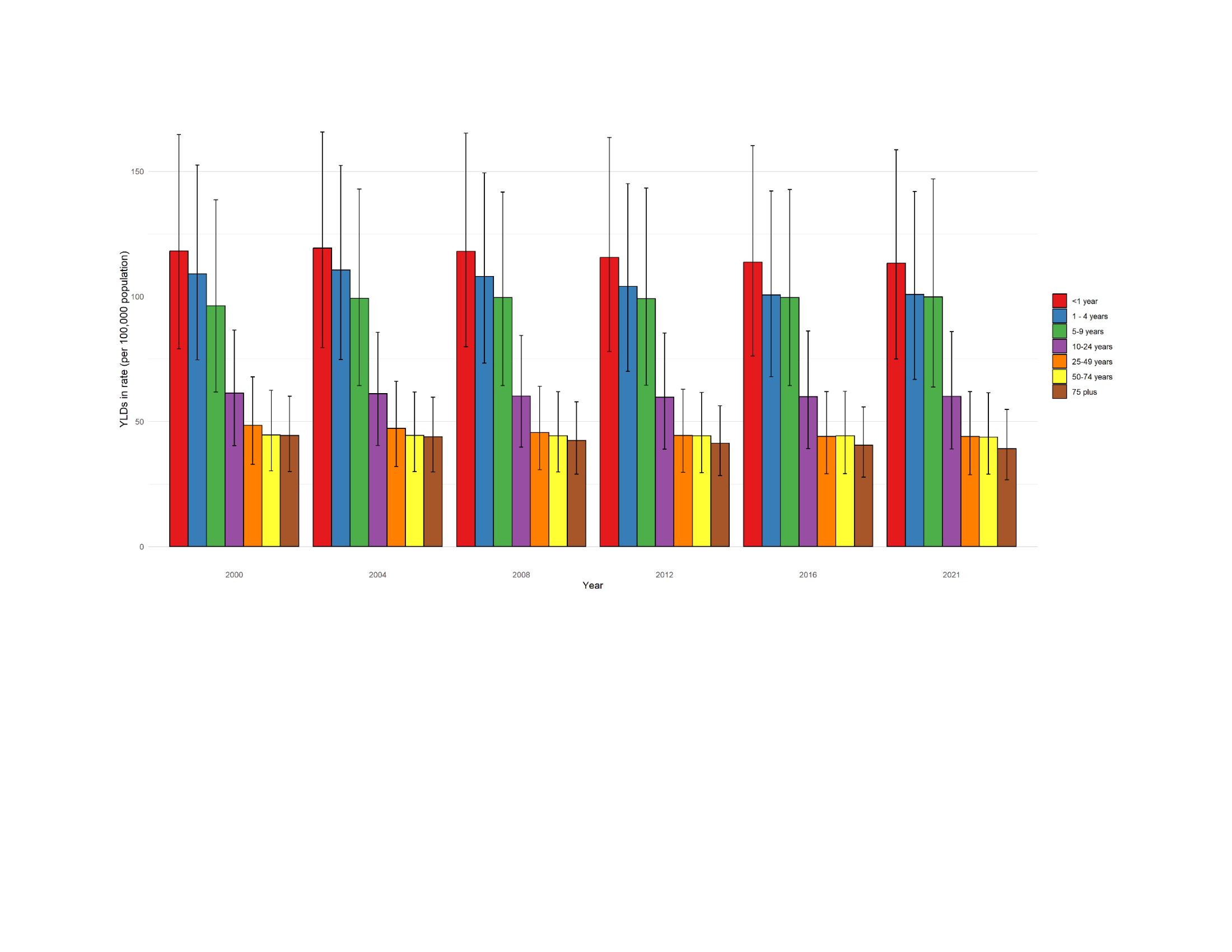

Supplement: S6 Fig — (TIFF) [file pgph.0005197.s006.tiff]

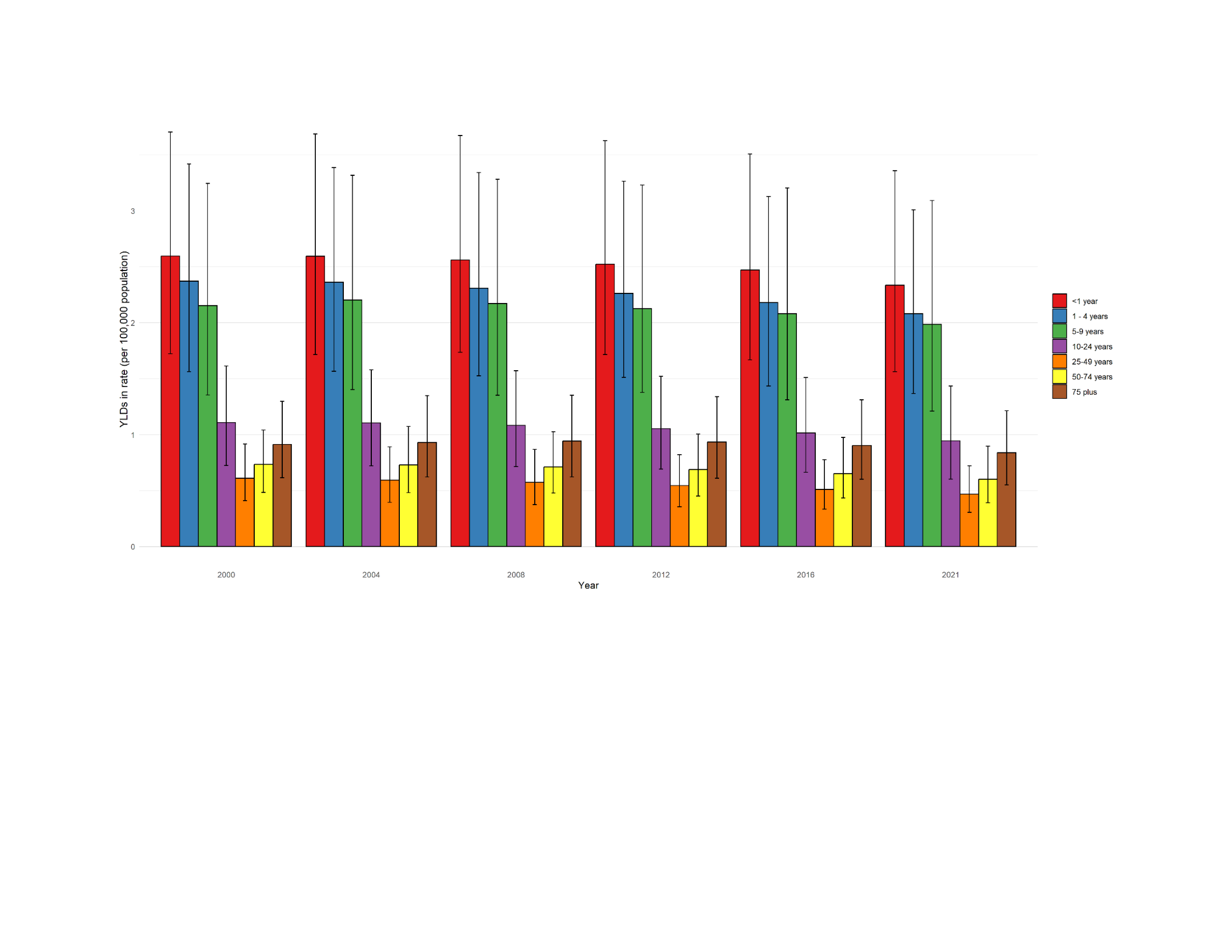

Supplement: S7 Fig — (TIFF) [file pgph.0005197.s007.tiff]

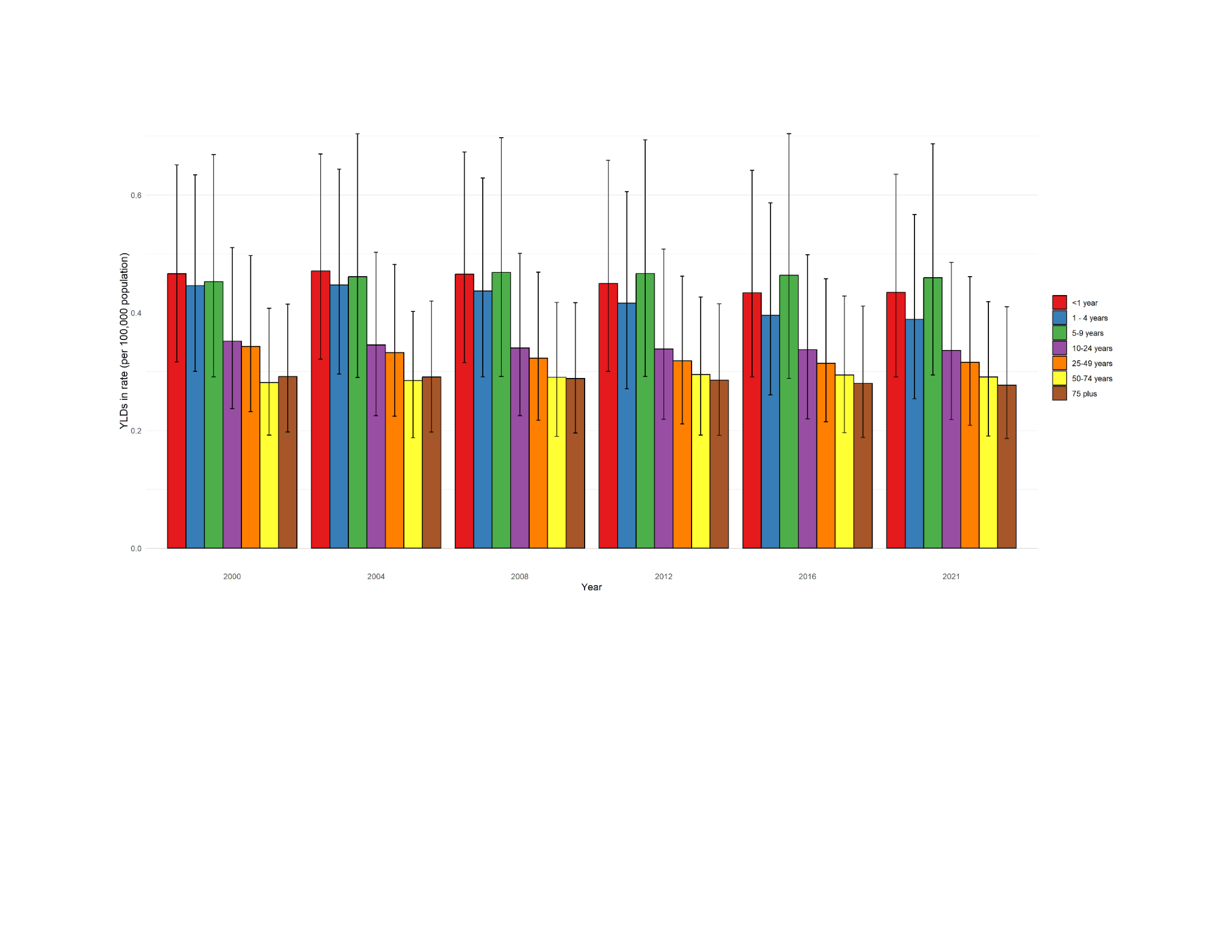

Supplement: S8 Fig — (TIFF) [file pgph.0005197.s008.tiff]

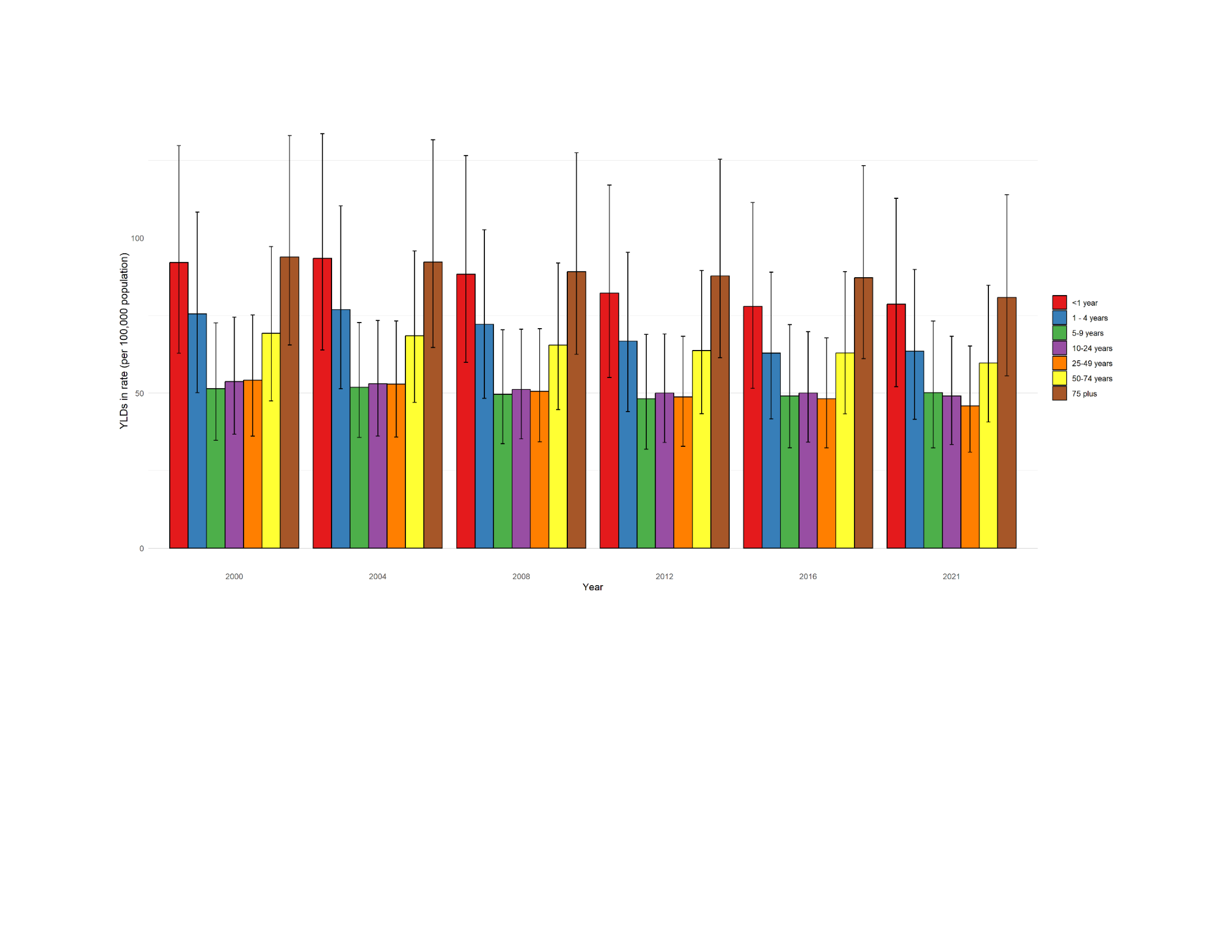

Supplement: S9 Fig — (TIFF) [file pgph.0005197.s009.tiff]
