# Supplementary material for: Burden of hemoglobinopathies and hemolytic anemias in the World Health Organization African region, 2000–2021: Findings from the Global Burden of Disease 2021 study
Source: PLOS Glob Public Health. 2025 Sep 22;5(9):e0005197. doi: 10.1371/journal.pgph.0005197 (PMC12453249; doi:10.1371/journal.pgph.0005197)
Supplement: Attachment — Submitted filename: PLOS GPH HHA_Response Letter_08062025_FINAL.docx [file pgph.0005197.s011.docx]

**Response Letter to Review Comments**

Title: Burden of hemoglobinopathies and hemolytic anemias in the World Health Organization African Region, 2000–2021: findings from the Global Burden of Disease 2021 Study

Submission/Review ID: PGPH-D-24-02847

Corresponding Author: Emmanuel Peprah (ep91@nyu.edu)

### ****Response to Reviewer Comments****

**Reviewer Critique 1: Does this manuscript meet PLOS Global Public Health’s publication criteria? Is the manuscript technically sound, and do the data support the conclusions? The manuscript must describe methodologically and ethically rigorous research with conclusions that are appropriately drawn based on the data presented.**

- **Reviewer #1:** Partly
- **Reviewer #2:** Yes

***Author Response:*** In response to Reviewer 1, we have revised and clarified the methodology section to better align with expectations for rigor and transparency. This manuscript uses publicly available data from the Global Burden of Disease (GBD) study, for which the corresponding author has served as a collaborator for over a decade. In line with the established norms of GBD publications, we refer readers to prior peer-reviewed GBD methodological papers for in-depth analytical techniques, while summarizing key aspects relevant to our analysis within the Methods section. We believe this approach enhances readability and maintains consistency with the broader body of GBD literature. References to core GBD methodological publications have been updated and expanded accordingly.

**Reviewer Critique 2: Has the statistical analysis been performed appropriately and rigorously?**

- **Reviewer #1:** No
- **Reviewer #2:** Yes

***Author Response:*** We appreciate Reviewer #1’s thoughtful concern regarding the rigor and clarity of our analytical procedures. In response, we have expanded the Methods section to provide additional detail and improve transparency. The foundational statistical analyses that support our findings were conducted by the Institute for Health Metrics and Evaluation (IHME), whose methodological framework forms the basis for all Global Burden of Disease (GBD) estimates. These methods are well-documented and have been rigorously peer-reviewed in prior GBD publications. To further enhance the specificity and relevance of our study, we independently accessed and analyzed publicly available GBD data for countries within the WHO African Region. These supplementary analyses were led by our co-author, Dr. Prince Amegbor (Assistant Professor at the New York University School of Global Public Health), an expert in geospatial analysis and environmental health statistics. Dr. Amegbor applied additional stratification and visualization techniques to contextualize regional variations and strengthen the robustness of our interpretations. These analytical steps are now explicitly described in the revised manuscript. We affirm that our findings are consistent with the GBD's established methodological framework and accurately reflect the regional burden of hemoglobinopathies and hemolytic anemias in the African context.

**Reviewer 1, Comment 1 (Abstract): The methods included in this section do not fully reflect the detailed methodology described in the main text. If the methods mentioned in the abstract are not aligned with those presented in the main section, both clarity and transparency may be compromised.**

***Author Response to Reviewer Comment 1:*** We thank the reviewer for this important observation regarding the alignment between the Abstract and the Methods section. We have revised the Abstract (Lines 32–39) to ensure greater consistency and transparency with the methodology described in the main text (Lines 93–116). The Abstract now succinctly reflects key aspects of the GBD methodology, including reference to analytical tools such as Bayesian meta-regression, and approaches for adjusting underreporting and misclassification of causes of death. While the Abstract offers a concise overview, the Methods section provides a more detailed summary with appropriate citations to peer-reviewed GBD publications and technical appendices. We have followed the standard GBD convention of referencing foundational methodological papers, as including the full set of analytical procedures (e.g., DisMod-MR 2.1, CODEm, YLD modeling) would significantly expand the manuscript length and potentially distract from the central findings. Our goal was to maintain both clarity and accessibility for a diverse readership. We hope the revised Abstract now offers an appropriate balance between brevity and methodological transparency, while the main Methods section guides readers to additional technical resources if further detail is needed.

**Reviewer 1, Comment 2 (Methods): The section is brief. It is recommended to include additional details about the methods used by the Global Burden of Disease (GBD) study to enhance reader understanding. Since GBD applies a complex methodology (e.g., Bayesian adjustments, redistribution of ill-defined causes), it is important to provide a minimal description of these key elements rather than relying solely on external references.**

***Author Response to Reviewer Comment 2:*** We sincerely appreciate Reviewer #1’s insightful suggestion to expand the Methods section for greater clarity and transparency. In response, we have revised the Methods section to include a more detailed yet concise summary of the core analytical approaches utilized by the GBD study. Specifically, we now describe the Cause of Death Ensemble model (CODEm), which integrates multiple predictive models and covariates to estimate cause-specific mortality with improved accuracy and precision. This model selects the best-performing algorithms based on predictive validity, ensuring robust mortality estimates across settings with variable data availability. Additionally, we have incorporated a description of DisMod-MR 2.1, the Bayesian meta-regression tool used by GBD to estimate incidence, prevalence, remission, and mortality in a statistically consistent manner. DisMod-MR 2.1 allows for the integration of disparate data sources, accounting for sampling error, measurement bias, and other uncertainties, while preserving internal consistency across disease parameters.

These methodological enhancements have been added to improve readers’ understanding of how GBD estimates are derived, without overwhelming the manuscript with technical detail. As such, we continue to reference the foundational GBD publications where the full methodological frameworks are comprehensively documented. We believe these revisions significantly enhance the transparency and rigor of the Methods section, in alignment with the reviewer’s helpful feedback.

**Reviewer 1, Comment 3 (Results): The statements in lines 136–137, 152–153, and 164–165 do not correspond to results; they appear to be comments that would be more appropriate in the discussion. It is suggested to relocate such sentences to the discussion section if they reflect interpretations or value judgments. The results section should focus exclusively on presenting findings objectively.**

***Author Response to Reviewer Comment 3:*** We thank the reviewer for this thoughtful and detailed feedback. In response, we have removed the content in lines 136–137, as it was interpretive in nature and better suited for the Discussion section. With regard to lines 152–153 and 164–165, we carefully reviewed these statements and believe they provide necessary contextualization and summarization of the data presented, without introducing subjective interpretation or value judgments. These lines serve to highlight key patterns and trends in the results, which we consider appropriate within the scope of a Results section. However, to ensure clarity and maintain an objective tone, we have made minor revisions to the wording of these statements to align more closely with a data-driven presentation. We hope these adjustments address the reviewer’s concern while preserving the informative value of the findings.

**Reviewer 1, Comment 4 (Results):**  **There is an inconsistency between line 215 (78.52) and Table 4 (78.62). The correct value should be verified and used consistently across the manuscript. Maintaining consistency between the text and tables is essential for analytical credibility.**

***Author Response to Reviewer 1 Comment 4:*** We thank the reviewer for this valuable feedback and finding this typo. The inconsistency between the value reported in line 215 and Table 4 has been corrected, and now consistently reflects the accurate value of 78.62 across all sections of the manuscript.

**Reviewer 1, Comment 5 (Results):** **Results should be standardized to one decimal place. It is recommended to harmonize the numerical presentation (ideally to one decimal place, unless greater precision is technically justified) throughout both text and tables.**

***Author Response to Reviewer 1 Comment 5:*** We thank the reviewer for the thoughtful and constructive comment regarding the standardization of numerical presentation. We fully agree that consistent formatting enhances readability and supports clearer interpretation of the findings. In response, we have revised all relevant tables to present values uniformly to two decimal places, in line with the reviewer’s suggestion. We appreciate the reviewer’s attention to this important aspect of scientific reporting.

**Reviewer 1 Comment 6 (Discussion):** **In line 241, specify that the reference is to children under 5 years of age, not under 4. The age group should be corrected to ensure accuracy.**

***Author Response to Reviewer 1 Comment 6:*** We thank the reviewer for this valuable feedback. The age group referenced in line 241 has been corrected to "children under 5 years of age" to ensure accuracy and consistency with the GBD reporting structure.

**Reviewer 1 Comment 7 (Discussion):** **The discussion could be enriched by comparing the findings with those from other studies and by providing explanations for the higher mortality observed in children under 5 and adults aged 75 and older. It is suggested to expand the discussion by including references from other regions or countries with similar profiles. Additionally, the findings should be interpreted in the context of structural factors such as demographic transition, access to healthcare services, and the burden of communicable versus non-communicable diseases.**

***Author Response to Reviewer 1 Comment 7*:** We have expanded the Discussion section to incorporate a comparative analysis with findings from other regions and relevant literature. Specifically, we highlight studies from sub-Saharan Africa, Southeast Asia, and Latin America that report similar mortality trends. In line with the reviewer’s recommendation, we also explore potential contributing factors, including health system capacity, socio-demographic transitions, and disparities in access to timely diagnosis and treatment.

**Reviewer 2 Comment 1: The article addresses a highly prevalent health burden in the WHO Africa region, 2000-2021 (i.e. that of DALYs and mortalities due to hemoglobinopathies and hemolytic anemias or HHAs). The manuscript is generally well-written but it is necessary to review it for clarity. The authors should be commended for the effort invested in coming up with their well-researched conclusions. However, one area that the authors of the article failed to address was the prevention of HHAs. As part of the conclusions/recommendations, I will suggest the authors include a statement or two on the provision or intensified use of targeted health education/counseling services e.g. pre-marital counseling, to further raise awareness about HHAs (particularly Sickle Cell Disease) in vulnerable communities across the WHO Africa region.**

***Author Response to Reviewer 2 Comment 1:*** We sincerely thank Reviewer 2 for the positive feedback and insightful suggestions. In response, we have thoroughly revised several key sections of the manuscript—including the Methods, Results, and Discussion—to improve clarity and alignment with the reviewer’s recommendations. Additionally, the Conclusion has been strengthened to underscore the critical importance of comprehensive screening strategies, public education, and access to effective treatment for HHA.

We thank the reviewers again for their thoughtful critique and believe that the revisions and clarifications we have provided significantly improve the clarity and quality of our manuscript. We remain enthusiastic about the opportunity to contribute to PLOS Global Public Health and look forward to your feedback.
